# PASNet: pathway-associated sparse deep neural network for prognosis prediction from high-throughput data

**DOI:** 10.1186/s12859-018-2500-z

**Published:** 2018-12-17

**Authors:** Jie Hao, Youngsoon Kim, Tae-Kyung Kim, Mingon Kang

**Affiliations:** 1 Kennesaw State University, Kennesaw, USA; 2 Kennesaw State University, Marietta, USA; 3 University of Texas Southwestern Medical Center, Dallas, USA; 4 Department of Life Sciences, Pohang Institute of Science and Technology (POSTECH), Dallas, USA

**Keywords:** Sparse deep neural network, Prognosis prediction, Long-term survival prediction, Pathway-based analysis, Glioblastoma multiforme, TCGA

## Abstract

**Background:**

Predicting prognosis in patients from large-scale genomic data is a fundamentally challenging problem in genomic medicine. However, the prognosis still remains poor in many diseases. The poor prognosis may be caused by high complexity of biological systems, where multiple biological components and their hierarchical relationships are involved. Moreover, it is challenging to develop robust computational solutions with high-dimension, low-sample size data.

**Results:**

In this study, we propose a Pathway-Associated Sparse Deep Neural Network (PASNet) that not only predicts patients’ prognoses but also describes complex biological processes regarding biological pathways for prognosis. PASNet models a multilayered, hierarchical biological system of genes and pathways to predict clinical outcomes by leveraging deep learning. The sparse solution of PASNet provides the capability of model interpretability that most conventional fully-connected neural networks lack. We applied PASNet for long-term survival prediction in Glioblastoma multiforme (GBM), which is a primary brain cancer that shows poor prognostic performance. The predictive performance of PASNet was evaluated with multiple cross-validation experiments. PASNet showed a higher Area Under the Curve (AUC) and F1-score than previous long-term survival prediction classifiers, and the significance of PASNet’s performance was assessed by Wilcoxon signed-rank test. Furthermore, the biological pathways, found in PASNet, were referred to as significant pathways in GBM in previous biology and medicine research.

**Conclusions:**

PASNet can describe the different biological systems of clinical outcomes for prognostic prediction as well as predicting prognosis more accurately than the current state-of-the-art methods. PASNet is the first pathway-based deep neural network that represents hierarchical representations of genes and pathways and their nonlinear effects, to the best of our knowledge. Additionally, PASNet would be promising due to its flexible model representation and interpretability, embodying the strengths of deep learning. The open-source code of PASNet is available at https://github.com/DataX-JieHao/PASNet.

## Background

Predicting prognosis in patients from large-scale genomic data is a fundamentally challenging problem in genomic medicine [1–3]. Along with the rapid advances of high-throughput technologies and their effectivenesses, high-dimensional genomic data provides more accurate and richer biological descriptions of clinical phenotypes of interests than ever before. Therefore, translating large-scale genomic profiles to clinical outcomes not only improves predicting patient prognosis but also helps in identifying prognostic factors and biological processes.

The capabilities of high-level biological representation and interpretation of the prognosis are often more desired in biomedical research rather than merely improving predictive performance. Pathway-based analysis is an approach that a number of studies have been investigating to improve both predictive performance and biological interpretability [4–6]. In pathway-based analyses, the incorporation of biological pathway databases in a model takes advantage of leveraging prior biological knowledge so that potential prognostic factors of well-known biological functionality can be identified. Pathway-based analyses identify biological links between pathways and clinical outcomes and enable the interpretation of biological processes where their corresponding genes and proteins are involved. Thus, pathway-based interpretation and visualization provide an intuitive and comprehensive understanding of functionally-related molecular mechanisms.

Moreover, pathway-based approaches have shown more reproducible analysis results than gene expression data analysis alone [4, 7–10]. High-level representations of gene co-expressions are considered in most pathway-based analyses; each of which represents a biological pathway while preserving the original information. Thus, pathway-based analyses remedy the limitations of gene expression data, which are intrinsically sensitive to stochastic fluctuations and are often caused by multiple potential sources, such as inherent stochasticity of biochemical processes, environmental differences, and genetic mutation [11]. Pathway-based markers were proposed for classifying breast cancer metastasis and ovarian cancer survival time [5]. Cancer subtypes were discovered with pathway-based markers via Restricted Boltzmann Machine (RBM) [8]. A group LASSO-based approach associated genes with pathways and characterized them based on biological pathways [10]. Higher-order functional representation of pathway-based metabolic features provided reproducible biomarkers for breast cancer diagnosis [9].

However, reliable and accurate prognosis still remains poor in many diseases due to the following challenges: high-dimension, low-sample size data and complex nonlinear effects between biological components.

Genomic data are highly dimensional relative to their sample sizes. High-dimension, low-sample size (HDLSS) data often make prediction models sensitive to noise and false positive associations, which consequently make predicting accurate prognoses difficult. LASSO-based approaches have been mainly considered to estimate the effects of a gene set that are associated with various types of clinical outcomes on HDLSS data. The LASSO-based approaches embed sparse coding schemes into linear or logistic regression models for selecting few but greatly informative features among the high-dimensional data. For instance, a logistic regression with sparse regularization was applied for the prognostic model of mortality after acute myocardial infarction [12]. Random LASSO was proposed to enhance the LASSO solution by applying multiple bootstrapping and was applied to predict patients’ survival times with glioblastoma gene expression data [13]. LASSO-based regression models as a prediction model were validated with multiple imputed data in chronic obstructive pulmonary disease patients [14].

Pathway-based analysis also helps to reduce data dimensionality. The number of biological pathways is relatively smaller than the number of genes, and a set of genes in the same pathway can be represented by the pathway’s effect. Thus, pathways can be used as summary variables for the input of the predictive model instead of including all genes, which consequently reduces the model complexity.

Most association studies between a gene set and various clinical outcomes have considered linear or logistic regression models for identifying prognostic factors as well as understanding a biological mechanism of the progression of disease. However, nonlinear effects of genes or pathways may fail to be identified by linear-based approaches. As a solution, kernel-based models have been proposed to capture nonlinear effects of complex pathways [15, 16]. Multiple kernel learning models were introduced to aggregate complex effects from multiple pathways [17, 18]. Kernel Principle Component Analysis (KPCA) was applied to reduce the dimensionality of the feature space by using the correlation structure of the pathways [18].

Recently, several attempts to capture hierarchical effects of genes and pathways have been made. Inferences of multilayered hierarchical gene regulatory networks have been considered to understand how pathways regulate each other hierarchically. A bottom-up graphic Gaussian model [19] and a recursive random forest algorithm [20] were proposed to construct multilayered hierarchical gene regulatory networks. Moreover, complex biological networks were modeled by inferring the multiple hierarchical models (1) between gene expression and pathways and (2) within pathways [21]. However, complex hierarchical relationships between pathways have not been considered for prognostic studies yet, to the best of our knowledge, although hierarchical effects of pathways are prevalent in biological systems [22].

In this paper, we propose a Pathway-Associated Sparse Deep Neural Network (PASNet) to achieve the goals: (1) to predict prognosis in patients accurately by incorporating biological pathways, (2) to provide a solution for hierarchical interpretation of nonlinear relationships between biological pathways of disease systematically, and (3) to handle computational problems on HDLSS data with unbalanced classes. An innovative aspect of our model is biological interpretability; we achieved this with sparse coding and by constructing hidden layers with biological pathways, which oppose the *black box* nature of deep learning. Our new sparse deep learning architecture represents multiple molecular biological layers, such as a gene layer and a pathway layer, along with their hierarchical relationships, which use sparse regularization.

## Results

Pathway-Associated Sparse Deep Neural Network (PASNet) identifies a subset of genes and pathways involved in a disease as prognostic biomarkers, as well as their interactions. PASNet models a multilayered, hierarchical biological system of genes and pathways on a disease, while leveraging the strengths of deep learning for competitive predictive performance. The sparsity of PASNet allows one to interpret the model, which is what conventional fully-connected networks lack. The architecture of PASNet and the strategies for training a sparse neural network model with HDLSS and imbalanced data are described in “Methods” section.

We conducted experiments to evaluate PASNet’s predictive performance for long-term survival prediction in Glioblastoma multiforme (GBM). The capability of the prediction was assessed by comparing our model with the classifiers that have been used for long-term survival prediction. Furthermore, we will describe how PASNet can represent the biological system of GBM in the following section.

### Data

GBM is a primary brain cancer that shows poor prognosis performance due to the above challenges. Comprising more than half of all brain tumors, GBM is the most prevailing and aggressive malignant type of primary astrocytomas [23]. Patients with GBM have a median survival time of approximately 15 months with intensive treatments [24]. Furthermore, long-term survival patients with GBM are rare as more than 90% of patients are deceased within three years of diagnosis. Although treatments in neurosurgery, chemotherapy, and radiotherapy have improved, the prognosis of GBM remains poor [25]. Hence, the advancement in understanding molecular mechanisms and related biological pathways of GBM is significant to accelerating the progress for new treatments [24].

We used the gene expression data of GBM patients, which is available at The Cancer Genome Atlas (TCGA, http://cancergenome.nih.gov). The dataset includes the gene expression data of 522 samples and 12,042 genes and provides survival time and status. We considered patients who survived past 24 months (regardless of survival status) as long-term survivals (LTS) and patients that deceased in less than 24 months as short-term survivals (non-LTS). Living patients with a survival time of less than 24 months were excluded in the experiments and considered censored data. Finally, we obtained 99 LTS and 376 non-LTS samples, where around 20% of the samples were LTS patients.

For pathway-based analysis, we utilized a biological pathway database from the Molecular Signatures Database (MSigDB) [26]. In MSigDB, we extracted the biological pathways of Reactome. Then, we excluded the pathways that include less than ten genes, because small pathways are often redundant with larger pathways. As the input features, we considered the genes that belong to at least one pathway, since pathway annotations of genes are essential to construct the mask matrix **M** between the gene layer and the pathway layer. Finally, we considered 574 pathways and 4359 genes in the experiments. The gene expression data were standardized to a mean of zero and a standard deviation of one.

### Experimental setting

We followed a typical design of conventional deep neural networks for PASNet. A sigmoid function and cross-entropy were considered for the activation and the cost function, respectively. A softmax function was used in the output layer so that the probabilities of output nodes add up to one. For the optimal tuning of PASNet’s training, we empirically determined the hyper-parameters by random search before cross-validation experiments. The learning rate (*η*) was set to 1e−4, and *L*^2^ regularization (*λ*) was set to 3e−4. Adaptive Moment Estimation (Adam) was performed as the stochastic optimizer [27]. The dropouts for two intermediate layers were also applied with a dropping probability of 0.8 and 0.7, respectively. PASNet was implemented by PyTorch, and the source code is available at https://github.com/DataX-JieHao/PASNet.

### Comparison

We evaluated PASNet by comparing the performance with classifiers that have been used for prognosis prediction: Support Vector Machine (SVM), Random LASSO [13], LASSO Logistic Regression (LLR) [1], and neural network with dropout (Dropout NN).

Specifically, we used a SVM with a radial basis function (RBF) kernel (*γ*=2^−16^ and *C*=2^3.9^ by two-step grid search [28]). Random LASSO was trained so that every feature could be selected 20 times on average by bootstrapping, and the *L*^1^ regularization parameter was determined by 10-fold cross-validation. The LASSO parameter for LLR was also selected by 10-fold cross-validation. The fully-connected Dropout NN was designed with the same numbers of intermediate layers and neurons as the proposed PASNet as well as the dropout probabilities. The learning rate was 0.01 and the *L*^2^ regularization was 0.005. Note that PASNet has less number of weights to be trained in each epoch because of sparse coding, compared to Dropout NN. Hence, the optimal hyper-parameters of *L*^2^ regularization and learning rate should be different between PASNet and Dropout NN. We empirically searched the optimal hyper-parameters for PASNet and Dropout NN separately through multiple experiments. Dropout NN was implemented by PyTorch (https://pytorch.org/).

The experiments were carried out by stratified 5-fold cross-validation for maintaining the same proportions of the imbalanced samples in the classes. The cross-validation experiments were repeated ten times for performance reproducibility. Data preprocessing, such as data normalization, was separately applied on each fold. The testing data on each fold was scaled with the mean and standard deviation of the training data of the same fold.

**Fig. 1:**
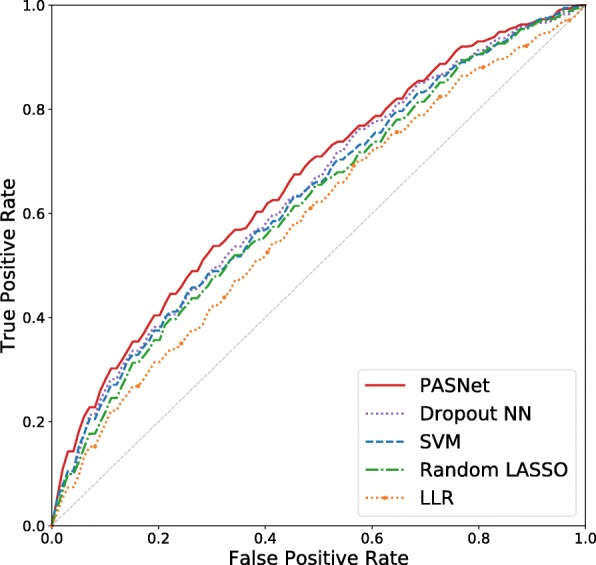
ROC Curves. PASNet produces the highest AUC of 0.6622 while the AUC of Dropout NN, SVM, random LASSO, and LLR is 0.6408, 0.6337, 0.6209, and 0.5899, respectively

The predictive performances of the five models were evaluated with two metrics: Area Under the Curve (AUC) and F1-scores. The Receiver Operating Characteristic (ROC) curve (see Fig. 1) was traced over the thresholds of scores to examine the trade-off between True Positive Rate (*TPR*=*TP*/(*TP*+*FN*)) and False Positive Rate (*FPR*=*FP*/(*FP*+*TN*)), where LTS was considered positive. An AUC was computed by the area under the ROC curve. An F1-score, an average of Positive Predicted Value (*PPV*=*TP*/(*TP*+*FP*)) and TPR, is calculated by 2(*PPV*×*TPR*)/(*PPV*+*TPR*). The F1-score was computed for the LTS class.

The average AUC and the average F1-score of the five methods on the test datasets are shown in Table 1. PASNet outperformed others as both AUC and F1-score are relatively high. PASNet produced AUC of 0.6622 ±0.013 (mean ±std) and F1-score of 0.3978 ±0.016. Following PASNet, Dropout NN produced AUC of 0.6408 ±0.014, and SVM produced AUC of 0.6337 ±0.015.

**Table 1:** Comparison of AUC and F1-score in over ten stratified 5-fold cross-validations

Model	AUC	F1-Score
Logistic LASSO	0.5899 ±0.020	0.3347 ±0.025
Random LASSO	0.6209 ±0.020	0.3370 ±0.020
SVM	0.6337 ±0.015	0.3446 ±0.015
Dropout NN	0.6408 ±0.014	0.2957 ±0.025
PASNet	0.6622 ±0.013	0.3978 ±0.016

To statistically assess the performance of PASNet (AUC) as compared to others, we conducted the Wilcoxon signed-rank test: a non-parametric paired, two sided test for the null hypothesis that states the median difference in paired samples is zero. Specifically, the null hypothesis is that the benchmark classifier has equal or better performance than our proposed algorithm. Table 2 shows the performance of PASNet is significantly better than others, where the null hypotheses are rejected at the 5% significance level (*p*-value <0.05). Hence, the outperformance of PASNet was statistically significant compared to the benchmark classifiers.

**Table 2:** The Wilcoxon signed-rank tests for comparing PASNet with the Benchmark Classifiers

	W Statistic	*P*-value
PASNet vs. Dropout NN	146.5	2.13e-06
PASNet vs. RBF-SVM	137.0	1.35e-06
PASNet vs. Random LASSO	45.0	1.06e-08
PASNet vs. Logistic LASSO	43.0	9.52e-09

SVM and Dropout NN showed a higher AUC than LASSO logistic regression and Random LASSO, probably because of their capability of capturing nonlinear effects of genes. Compared to Dropout NN, PASNet is a relatively thin network, where the connections between layers are very sparse. However, PASNet interestingly produced higher performance than Dropout NN. It shows that PASNet builds a robust network model, which is simplified to represent the biological processes for prognosis prediction by incorporating biological prior knowledge.

## Discussion

**Fig. 2:**
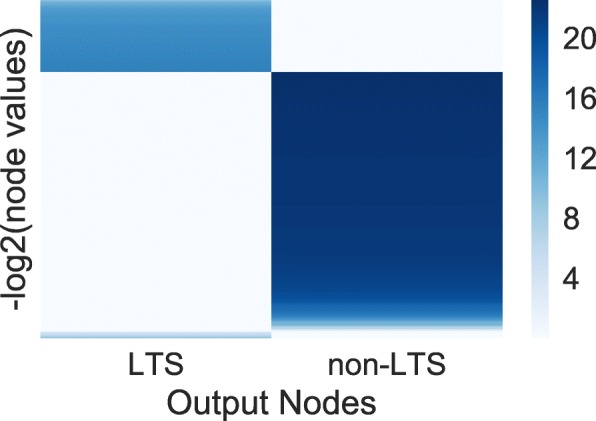
Graphical representation of the output node values over the samples by PASNet. LTS samples obtain higher node values in LTS node than non-LTS samples. Similarly, non-LTS samples obtain higher node values in non-LTS node than LTS samples

**Fig. 3:**
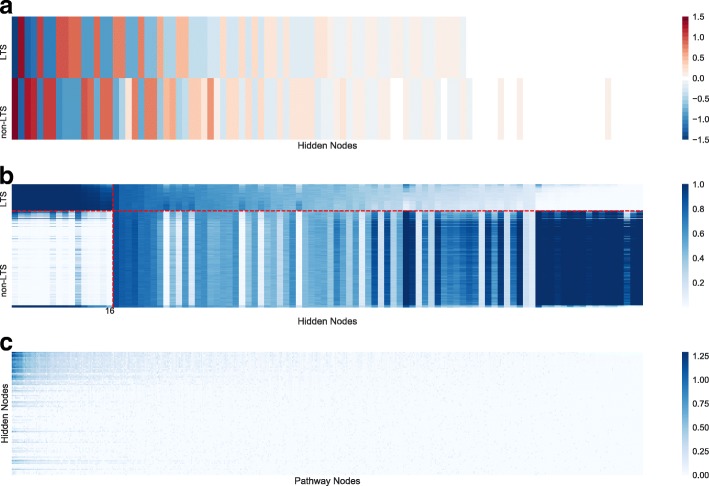
Graphical representation among the output layer, hidden layer, and pathway layer in PASNet. (**a**) The weights between the hidden layer and the output layer. Hidden nodes are sorted in a descending order. (**b**) The node values in the hidden layer. The horizontal dotted lines indicates LTS/non-LTS samples. The vertical dotted lines indicates LTS/non-LTS samples are significantly distinguished by top 16 pathways. (**c**) The absolute weights between the pathway layer and the hidden layer

**Fig. 4:**
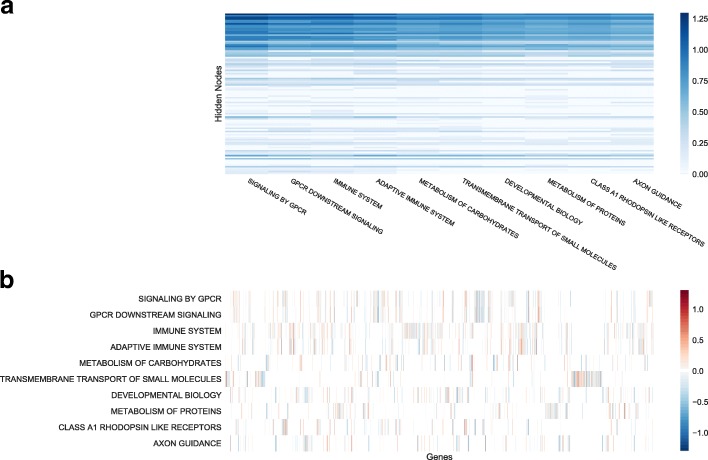
Graphical representation of the 10 top-ranked pathways by PASNet (**a**) The absolute weights between the 10 top-ranked pathway nodes and the hidden layer. It is a zoom-in view of Fig. 3c. (**b**) Weights between the gene layer and the 10 top-ranked pathway nodes. The connections are determined by Reactome database

Although PASNet yielded competitive predictive performance in the experiments, a more promising contribution of PASNet is in the model’s interpretability. In this section, we demonstrate a plausible biological mechanism inferred by PASNet for long-term survival prediction in GBM. The graphical representations of the PASNet model are illustrated in Figs. 2, 3 and 4 in the top-down order. The heatmaps were generated by sorting the weights and node values of LTS, and positive and negative weight values are colored in red and blue, respectively.

First, Fig. 2 manifests the posterior probability of the samples in the clinical outcomes. The dark block on the top shows the output node values (−*log*_2_(node value)) of the LTS samples, while the remaining ones are non-LTS samples. The weight values of the connections from hidden nodes to the output nodes are depicted in Fig. 3a, where dropped connections are colored in white. The figure reveals distinct patterns of weights (opposite signs) to the two output neurons. Note that there are hidden nodes disconnected to the neurons in the output layer (colored in white) by sparse coding, which shows that the hidden nodes are insignificant.

The hidden node values of the samples are shown in Fig. 3b. The values of the hidden nodes indicate the intensity of the group effects on the pathways, which are connected to the hidden nodes. For instance, the first 16 hidden nodes in Fig. 3b show distinguishable intensities on LTS and non-LTS patients. The LTS patients present significant intensities of the group effects of the 16 pathways while non-LTS patients show significant lower values.

The weights between the pathway nodes and the hidden nodes are exhibited in Fig. 3c, and the top-10 ranked pathways among them are zoomed in Fig. 4a. It appears that a small number of pathways mainly contribute to the hidden nodes simultaneously, which implies that the cohort of the pathways may be candidates of prognostic biomarkers in long-term survival of GBM. The top-10 ranked pathways include signaling by GPCR, GPCR downstream signaling, innate immune system, adaptive immune system, metabolism of carbohydrates, transmembrane transport of small molecules, developmental biology, metabolism of proteins, class A/1 (rhodopsin-like receptors), and axon guidance. Most of the pathways are referred to as significant pathways in GBM in biological literature. The pathways and the references are listed in Table 3. Since the top-10 ranked pathways are all large (gene numbers >200), we further explored small pathways as well. Class B/2 (Secretin family receptors) pathway which includes 88 genes is ranked 14th. One of the subgroups in Class B/2 family is categorized as brain-specific angiogenesis inhibitors that are growth suppressors of glioblastoma cells [29]. Hence, Class B/2 pathway may play an important role in inhibition of GBM.

**Table 3:** Top-10 ranked pathways for survival prediction in GBM by PASNet

Pathway name	Pathway size	Reference	Top-5 ranked genes^a^
Signaling by GPCR	920	[33]	SHH, PTGFR, GNG5, CHRM5, LHB
GPCR downstream signaling	805	[50]	PTGFR, OR7C2, GNG5, OR10H3, MLNR
Innate immune system	933	[35]	CD79B, INPPL1, SRC, NUP85, DNM2
Adaptive immune system	539	[51]	CD79B, ASB6, PTEN, NCF4, FBXO2
Metabolism of carbohydrates	247	-	HS3ST3B1, NUP85, PFKFB3, LUM, SLC2A4
Transmembrane transport of small molecules	413	[52]	SLC9A7, ABCA7, GNG5, AQP8, HK3
Developmental biology	396	-	NRP2, FES, WNT10B, MYOD1, SLC2A4
Metabolism of proteins	518	-	EIF3G, CCT2, TIMM22, RPL3L, GMPPA
Class A/1 (rhodopsin-like receptors)	305	[53]	PTGFR, OPRD1, CHRM5, NPFF, NTSR2
Axon guidance	251	[54]	NRP2, NRTN, AGRN, FES, RPS6KA4

^a^The genes were ranked by absolute weights in the pathways

The genes of the pathways are illustrated by the weight values in Fig. 4b. Since the connections between the gene layer and the pathway layer are given by pathway databases, e.g., Reactome, they are very sparse. It also shows that multiple pathways share genes in common. The genes, which are most frequently shown in the ten pathways, include CDC42, PRKCQ, RAC1, AKT1, AKT2, AKT3, C3, CREB1, GRB2, HRAS, KRAS, NRAS, PRKACA, PRKACB, PRKACG, RAF1, and YWHAB, where CDC42, PRKCQ, and RAC1 are shown in six pathways and others are in five pathways. Among them, several genes have been reported as biomarkers in GBM. For instance, AKT1, AKT2, and AKT3, belonging to the five pathways of signaling by GPCR, GPCR downstream signaling, innate immune system, adaptive immune system, and developmental biology, are three isoforms of AKT in PI3K/AKT pathway, which is an important drug target in many cancers including GBM [30]. In particular, AKT2 is a well-known proto-oncogene that promotes the growth of tumors and reduces the survival of patients in GBM [31, 32].

**Fig. 5:**
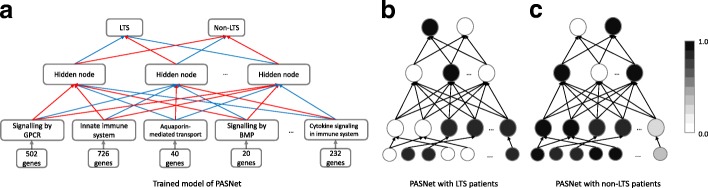
Hierarchical representation of pathways in PASNet. (**a**) PASNet is partially visualized showing the five pathways. Distinct neural network activations between LTS (**b**) and non-LTS (**c**) are shown via PASNet. The nodes of the neural network of (b) and (c) correspond to (a). For instance, the nodes in the pathway layer of (b) and (c) represent signaling by GPCR, innate immune system, aquaporin-mediated transport, signaling by BMP, and Cytokine signaling in immune system. The pathways of signaling by GPCR and innate immune system are inactive with LTS patients, whereas the both pathways are active with non-LTS patients

Finally, we demonstrate a hierarchical representation of genes and pathways in PASNet. In Fig. 5a, PASNet is partially visualized, where positive and negative weights are colored in red and blue respectively. The pathways are represented by the corresponding genes in the pathway layer, and then the nonlinear effects of the pathways are described in the hidden layer. The hierarchical representations can be captured in the output layer, which produces a posterior probability for prognosis prediction. Although we considered a single hidden layer to simplify the model with HDLSS data in this study, multiple hidden layers may be able to capture the biological processes and their effects more accurately if a sufficient number of samples are available. Figure 5b–c illustrate distinctive representations of LTS and non-LTS samples in PASNet. The color of nodes in the figures shows the values computed with LTS/non-LTS samples in average. Note that node values between the pathway layer and the output layer are between zero and one. The node with a high value may be a potential prognostic biomarker in the group. Figure 5b shows that pathways including aquaporin-mediated transport, signaling by BMP, and cytokine signaling in immune system are activated with LTS samples. The second node in the hidden layer is triggered by the active pathways, and the hidden node activates the LTS node in the output layer. On the other hand, Fig. 5c shows that additional pathways of signaling by GPCR and innate immune system are also activated for non-LTS samples. The other two hidden nodes take the active pathways into account, and they activate the non-LTS node in the output layer. Hence, the two pathways of signaling by GPCR and innate immune system may be potential prognostic biomarkers for predicting LTS/non-LTS. Pathway of signaling by GPCR has been investigated as a potential therapeutic target to inhibit the progression of glioblastomas. [33]. Activating the innate immune system, i.e. immunotherapy, is a promising strategy for the treatment of GBM [34]. Vascular endothelial growth factor (VEGF), a modulator of the innate immune system, is reported crucial for the tumor progression [35]. Moreover, aquaporin-mediated transport, signaling by BMP, and cytokine signaling in immune system may play an important role in GBM, since they are shown in common as active in both LTS and non-LTS. Note that the activation/inactivation of a node in PASNet does not directly represent biological activation in the system, whereas it indicates different states of the biological components in the groups.

## Conclusions

In this paper, we proposed pathway-associated sparse deep neural network for prognosis predictions (long-term survivals in GBM in this study). PASNet builds a network model by leveraging prior biological knowledge of pathway databases and by taking hierarchical nonlinear relationships of biological processes into account. To improve the model interpretability, PASNet introduces sparse coding. Moreover, we developed a training strategy to avoid the overfitting problem with HDLSS data and the imbalanced problem.

To investigate the performance of PASNet, we used gene expression data of GBM patients in TCGA. PASNet was assessed by comparing the predictive performance with support vector machine, random LASSO, LASSO logistic Regression, and neural network with dropout that have been widely used for prognosis prediction. PASNet outperformed them with respect to both AUC and F1-score in the multiple stratified 5-fold cross-validation experiments. Furthermore, we discussed how PASNet can describe the biological system of GBM.

PASNet is the first deep neural network-based model that represents hierarchical representations of genes and pathways and their nonlinear effects, to the best of our knowledge. Additionally, PASNet would be promising due to its flexible model representation and interpretability, embodying the strengths of deep learning.

## Methods

### The architecture of PASNet

**Fig. 6:**
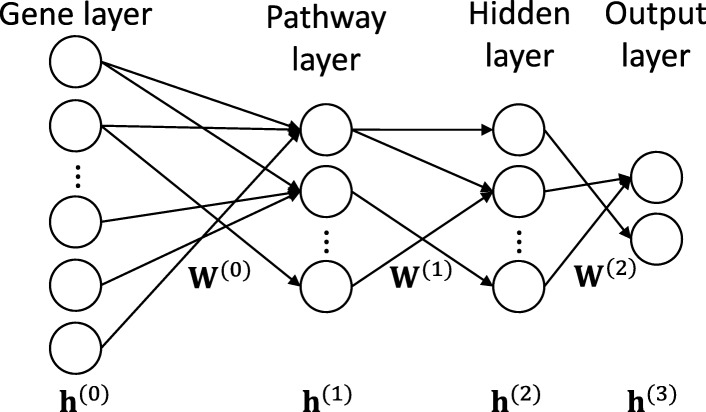
Architecture of PASNet. The structure of PASNet is constructed by a gene layer (an input layer), a pathway layer that represents the biological pathways linked with input genes, a hidden layer that represents hierarchical relationships among biological pathways, and an output layer that corresponds with clinical outcomes, e.g. a binary class that has long-term survival and short-term survival, stages of cancer

PASNet incorporates biological pathways and the concept of sparse modeling based on Deep Neural Network (DNN). The neural network architecture of PASNet consists of a gene layer (an input layer), a pathway layer that represents the biological pathways linked with input genes, a hidden layer that represents hierarchical relationships among biological pathways, and an output layer that corresponds with clinical outcomes, e.g. a binary class that has long-term survival and short-term survival, stages of cancer (see Fig. 6).

In PASNet, sparse coding is considered on the connections between layers for model interpretability. Sparse coding provides a solution to capture significant components of a biological mechanism in the model, since biological processes may involve only a few biological components. On the other hand, conventional fully-connected networks lack to represent biological mechanisms.

#### Gene layer

The gene layer (as an input layer) corresponds to gene expression data. A patient sample of *m* gene expressions is formed as a column vector, which is denoted by **x**={*x*_1_,*x*_2_,...,*x*_*m*_}. Each input node represents one gene feature.

#### Pathway layer

The pathway layer represents biological pathways, where each node indicates an individual pathway. The connections between the gene layer and the pathway layer are established by well-known pathway databases (e.g., Reactome and KEGG). Pathway databases contain associations between pathways and genes; each of which provides a set of gene components. Therefore, the pathway layer makes it possible to interpret the model as a pathway-based analysis.

To begin with initializing the connections between the gene layer and the pathway layer, we consider a binary biadjacency matrix (**A**) from biological pathway databases. The biadjacency matrix can be defined as $\textbf {A} \in \mathbb {B}^{n \times m}$, where *n* is number of pathways and *m* is number of genes. Then, an element of **A**, i.e., *a*_*ij*_, is set to one if gene *j* belongs to pathway *i*; otherwise, zero. Sparse coding is applied based on the matrix **A** to represent the relationships between genes and pathways in the model.

#### Hidden layer

Biological components may cooperate with others instead of functioning alone. A biological system involves multiple pathways which have interactions together, whereas a node in the pathway layer indicates a biological pathway. The associative interactions between pathways can be represented in the hidden layer. In PASNet, the hidden layer represents biological nonlinear associations between the pathways to outputs.

Sparse coding between the pathway and the hidden layers enables one to interpret these relationships. Although we consider only a single hidden layer in this study for simplicity’s sake, multiple hidden layers can be used for deeper hierarchical representations of pathways. For example, if there are two hidden layers, the second hidden layer will represent deeper hierarchical associations of the nodes of the first hidden layer, which are association effects of pathways.

#### Output layer

The output layer shows clinical outcomes for which nodes compute the posterior probabilities. In this layer, sparse coding allows to distinguish hierarchical groups of pathways (which are detected from hidden layers) to predict clinical outcomes. In PASNet, more than two clinical outcomes can be easily represented with multiple nodes in the output layer.

Consequently, PASNet can dissect distinguishable biological processes of hierarchical nonlinear relationships and associations of genes and pathways to predict clinical outcomes. Furthermore, this *generative* model-based approach would be often useful to predict prognosis accurately with complex data of HDLSS. When data is highly complex and only small sample sizes are available, model optimization may be easily biased to the training data rather than providing a general solution. On the other hand, the integration of the biological structures and prior knowledge to the model would produce a robust solution.

### Overall description of PASNet training

**Fig. 7:**
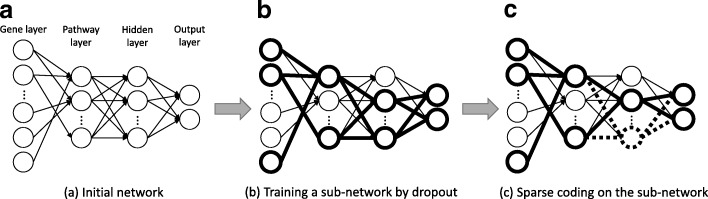
Training of PASNet. (**a**) Weights and biases are randomly initialized. Connections between the gene layer and the pathway layer are determined by biological pathway databases, and the remaining layers are considered as fully-connected in this step. (**b**) A sub-network is randomly selected using a dropout technique and trained. (**c**) Sparse coding optimizes the sparsity of connections in the sub-network

The main challenge in training PASNet is to reduce both risk of overfitting and computational complexity of training on HDLSS data. The related works that have handled the HDLSS data problem are discussed in “Related works in deep learning” section. To unravel the problems, PASNet optimizes a small sub-network, which involves feasible nodes and parameters to train instead of the whole network and then makes the sub-network sparse. Figure 7 illustrates the overall training flow of PASNet.

First, we initialize the connections between the gene layer and the pathway layer with prior biological knowledge of pathways (see Fig. 7a). Active/inactive connections are determined by the biadjacency matrix, **A**. The weights of active connections and biases are randomly initialized from standard normal distribution, while the weights of inactive connections are set to zero. The sparsity of the connections between the gene layer and the pathway layer is invariant over the entire training. The remaining layers are fully interconnected as the initial.

In the training phase, we repeat training sub-networks and applying sparse coding on the sub-networks until convergence (Fig. 7b–c). A sub-network is selected by a dropout technique, where neurons are randomly dropped in the intermediate layers. In Fig. 7b, a small sub-network is shown with bold solid circles and lines. Then, the small sub-network is trained by feed-forward and backpropagation. Note that only weights and biases of the sub-network are trained. Upon the completion of the sub-network’s training, sparse coding is applied to the sub-network by trimming the connections that do not contribute or worsen to minimize the loss. In Fig. 7c, the dropped connections and nodes are marked as bold, dashed lines. The details of the training are elucidated in the following sections.

### Sparse coding

Once a small sub-network is completed to train with the HDLSS data, the sub-network is imposed to be sparse for the model interpretation. The sparsity of the sub-network is determined by the mask matrix **M** on each layer as:

1 $$ \textbf{h}^{(\ell +1)} = a\left(\left(\textbf{W}^{(\ell)}\star\textbf{M}^{(\ell)}\right)\textbf{h}^{(\ell)}+\textbf{b}^{(\ell)}\right),   $$

where ⋆ denotes element-wise multiplication, and *a*(·) is an activation function. **h**^(*ℓ*)^ denotes an output vector on the *ℓ*-th layer, and **W**^(*ℓ*)^ and **b**^(*ℓ*)^ are a weight matrix and a bias vector, respectively. An element value of **M** is either one or zero, which determines whether the associated weights are dropped in the current epoch.

The mask matrix **M** is generated with respect to a sparsity level (*S*) that indicates the proportion of weights to be dropped in a single layer. *S* is a value between 0 to 100, where zero creates a fully-connected layer while 100 causes no connection. The optimal *S*^∗^ is approximated on each layer individually in the sub-network, while most related methods consider a single hyper-parameter for the sparsity of all layers [36, 37]. The individual setting of the sparsity on each layer shows different levels of biological associations on the genes and pathways.

We obtain the optimal sparsity level *S*^∗^ that minimizes the cost score. For efficient computation, the cost scores are computed with a small number of finite sparsity levels. Then, the optimal sparsity level is estimated by applying a cubic-spline interpolation to the cost scores with the assumption that the cost function, with respect to the sparsity level, is continuous.

In particular, an element of **M** is set to one if the absolute value of the corresponding weight is greater than threshold *Q*; otherwise, the element is zero, where *Q* is an *S*-th percentile of absolute values of **W**. Note that the mask between the gene layer and the pathway layer, i.e. **M**^(0)^, is determined by the biadjacency matrix **A** of biological pathways. Thus, the mask matrices are formulated as

2 $$ \textbf{M}^{(\ell)}= \begin{cases} \mathbbm{1}\left(\lvert \textbf{W}^{(\ell)} \rvert \geq Q^{(\ell)}\right), & \text{if}\, \ell \neq 0 \\ \textbf{A}, & \text{if}\, \ell = 0 \end{cases}   $$

where *Q*^(*ℓ*)^ is the *S*-th percentile of |**W**^(*ℓ*)^| if *ℓ*≠0.

### Cost-sensitive learning for imbalanced data

We refine the cost function and the backpropagation for cost-sensitive learning, since imbalanced data causes bias of the predictions towards the majority class. We adapt the Mean False Error (MFE) method [38], which penalizes the errors of the majority class.

Let *K* be the number of clinical outcomes. The normalized cost is computed separately for each class by:

3 $$ \mathcal{L} = \sum\limits_{k=1}^{K} \mathcal{C}_{k} + \frac{1}{2}\lambda\lVert \textbf{W}\lVert_{2},   $$

4 $$ \mathcal{C}_{k} = \frac{1}{n_{k}}\sum\limits_{i=1}^{n_{k}}c\left(\textbf{y}_{i}, \tilde{\textbf{y}}_{i}\right),  $$

where $\mathcal {C}_{k}$ denotes mean error on the class *k*, and *n*_*k*_ is the number of samples in the class *k*. **y**_*i*_ is a vectorized ground truth class label of the *i*-th sample, and $\tilde {\textbf {y}}_{i}$ is its vectorized prediction. *c*(·) denotes a cost function (e.g., cross-entropy loss), and $\mathcal {L}$ is the total cost. ∥**W**∥_2_ denotes a *L*^2^-norm of **W**, and *λ*>0 is a regularization hyperparameter.

In the backpropagation phrase, the gradient is also computed separately for each class. Hence, the weights and biases on the *ℓ*-th layer are updated by:

5 $$\begin{array}{@{}rcl@{}} \textbf{W}^{(\ell)} &\leftarrow& (1-\eta\lambda)\textbf{W}^{(\ell)} - \eta\sum\limits_{k=1}^{K} \frac{\partial \mathcal{C}_{k}}{\partial \textbf{W}^{(\ell)}},  \end{array} $$

6 $$\begin{array}{@{}rcl@{}} \textbf{b}^{(\ell)} &\leftarrow& \textbf{b}^{(\ell)} - \eta\sum\limits_{k=1}^{K} \frac{\partial \mathcal{C}_{k}}{\partial \textbf{b}^{(\ell)}},  \end{array} $$

where *η* is a learning rate. The algorithm of PASNet is briefly described in Algorithm 1.


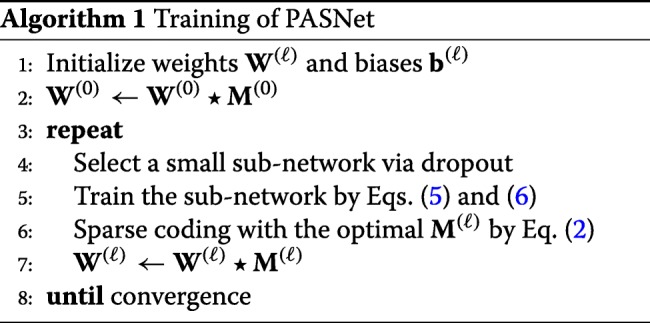


## Related works in deep learning

In recent years, deep learning has been spotlighted as the most active research field in various machine learning communities, such as image analysis, speech recognition, and natural language processing as its promising potential is being actively discussed in bioinformatics and biomedicine [39]. Most deep learning-based approaches have been developed for classification and association studies in bioinformatics. For instance, D-GEX infers the expression of target genes from landmark genes, capturing the nonlinear relationships by combining gene expression, DNA methylation, and miRNA expression data [40]. A convolutional neural network (CNN) was adapted to predict DNA-protein binding sites with Chromatin Immunoprecipitation sequencing (ChIP-seq) data [41]. Additionally, CNN-based DeepBind was proposed to predict whether a specific DNA/RNA binding protein will bind to a specific DNA sequence [42]. The functionality of non-coding variants was predicted by DeepSEA by employing a CNN model [43].

Although only a small subset of deep learning research has been reported in bioinformatics due to the difficulty of structure definition and interpretation, the future of deep learning in biology and medicine is promising [44]. First, since a neural network is inspired by the neurons in the human brain, a neuron network architecture is applicable to modeling a mechanism for a complex biological system. Specifically, deep learning approaches take advantage of flexible representation of hierarchical structures from inputs to outputs. The representation of nonlinear effects of neurons in multiple layers in neural networks may be able to model hierarchical biological signals. DCell constructs a multi-layer neural network based on extensive prior biological knowledge to simulate the growth of a eukaryotic cell [45]. However, DCell’s network architecture is entirely based on well-known prior biological knowledge, so the model was applied to relatively simple biological system of yeast. Moreover, deep learning captures nonlinear effects of variables with high-level feature representation, which allows deep learning to outperform other state-of-the-art methods.

However, training deep neural networks with HDLSS data poses a computational problem. A large number of parameters are involved in deep neural networks, and it often makes the training infeasible or causes a model overfit on HDLSS data. Particularly, backpropagation gradients in neural networks are of high variance on HDLSS data, which consequently causes the model overfit [46]. In order to tackle the HDLSS problem, the leave-one-out approach was used to avoid the overfitting problem in backpropagation [47]. Regarding backpropagation, the risk of overfitting was examined with validation data by the leave-one-out approach and terminates the training early when overfitting occurs. For an alternative solution, an attempt to reduce the dimensionality of the input space to a feasible size has been made [48]. Dimension reduction techniques, such as subsampled randomized Hadamard transform (SRHT) and Count Sketch-base construction, were utilized to reduce the dimensional size of the input data. Then, the projected data into the lower space were introduced to a neural network for training.

For HDLSS data, feature selection is one of the conventional approaches. Deep Feature Selection (DFS) was developed to select a discriminative feature subset in a deep learning model [49]. Although DFS is not the optimal solution to low-sample size data, DFS shows that deep learning can detect informative and discriminative features of nonlinearity effects through multiple layers with high-dimensional data. Then, Deep Neural Pursuit (DNP) improved the solution of the feature selection in deep learning, taking the HDLSS data problem into account [46]. DNP iteratively augments features in the input layer by performing multiple dropouts. The multiple dropouts grant the ability to train a small-sized sub-network at a time and to compute gradients with low variance for alleviating the overfitting problem.

## Abbreviations

Adam: Adaptive moment estimation
AUC: Area under the curve
ChIP-seq: Chromatin Immunoprecipitation sequencing
CNN: Convolutional neural network
DFS: Deep feature selection
DNN: Deep neural network
DNP: Deep neural pursuit
Dropout NN: Neural network with dropout
GBM: Glioblastoma multiforme
HDLSS: High-dimension, low-sample size
KPCA: Kernel principle component analysis
LLR: LASSO logistic regression
MFE: Mean false error
MSigDB: Molecular signatures database
PASNet: Pathway-associated sparse deep neural network
RBF: Radial basis function
RBM: Restricted Boltzmann machine
ROC: Receiver operating characteristic
SRHT: Subsampled randomized hadamard transform
SVM: Support vector machine
TCGA: The cancer genome atlas
